# Top-Down Crawl: a method for the ultra-rapid and motif-free alignment of sequences with associated binding metrics

**DOI:** 10.1093/bioinformatics/btac653

**Published:** 2022-09-30

**Authors:** Brendon H Cooper, Tsu-Pei Chiu, Remo Rohs

**Affiliations:** Department of Quantitative and Computational Biology, University of Southern California, Los Angeles, CA 90089, USA; Department of Quantitative and Computational Biology, University of Southern California, Los Angeles, CA 90089, USA; Department of Quantitative and Computational Biology, University of Southern California, Los Angeles, CA 90089, USA; Departments of Chemistry, Physics & Astronomy, and Computer Science, University of Southern California, Los Angeles, CA 90089, USA

## Abstract

**Summary:**

Several high-throughput protein–DNA binding methods currently available produce highly reproducible measurements of binding affinity at the level of the *k-*mer. However, understanding where a *k-*mer is positioned along a binding site sequence depends on alignment. Here, we present Top-Down Crawl (TDC), an ultra-rapid tool designed for the alignment of *k-*mer level data in a rank-dependent and position weight matrix (PWM)-independent manner. As the framework only depends on the rank of the input, the method can accept input from many types of experiments (protein binding microarray, SELEX-seq, SMiLE-seq, etc.) without the need for specialized parameterization. Measuring the performance of the alignment using multiple linear regression with 5-fold cross-validation, we find TDC to perform as well as or better than computationally expensive PWM-based methods.

**Availability and implementation:**

TDC can be run online at https://topdowncrawl.usc.edu or locally as a python package available through pip at https://pypi.org/project/TopDownCrawl.

**Supplementary information:**

Supplementary data are available at *Bioinformatics* online.

## 1 Introduction

High-throughput *in vitro* binding methods, such as protein binding microarrays (Berger *et al.*, 2006), SELEX-seq (Riley *et al.*, 2014; Slattery *et al.*, 2011) and SMiLE-seq (Isakova *et al.*, 2017), have given researchers the ability to precisely quantify transcription factor (TF) binding in a controlled environment using unbiased pools of DNA. For each of these methods, the enrichment of each individual probe is not as informative as the enrichment of *k-*mers. While each full-length probe may occur a few times within a sample, shorter *k-*mers will occur more frequently, providing highly reproducible measures of binding affinity. Since *k-*mer enrichment is inherently context-free, it is common to see a high level of enrichment for *k-*mers that only covers a portion of the binding site. Determining which part of the binding site a *k-*mer covers depends on alignment. Alignment allows researchers to pinpoint TF–DNA interactions along the binding site and is a necessary step in the application of conventional machine learning approaches such as multiple linear regression (MLR). Previously described approaches, such as MEME (Bailey & Elkan, 1994), BEESEM (Ruan *et al.*, 2017) and SelexGLM (Zhang *et al.*, 2018) are designed to generate position weight matrices (PWMs) which can subsequently be used to align *k-*mer level data, but they were not developed for this purpose. Furthermore, using a PWM to summarize binding preferences for a TF is an unnecessary abstraction from the original *k-*mer level data and results in the loss of information regarding interdependencies between positions of the binding site. It is already known that DNA shape is dependent on local interactions across several base pairs (bp) and plays a significant role in protein–DNA binding for many TFs (Yang *et al.*, 2017). Here, we describe a new approach called Top-Down Crawl (TDC), which can rapidly align large sets of *k-*mer level quantitative binding data in a rank-dependent manner that does not depend on experiment-specific parameterization.

## 2 TDC implementation

**Fig. 1.**
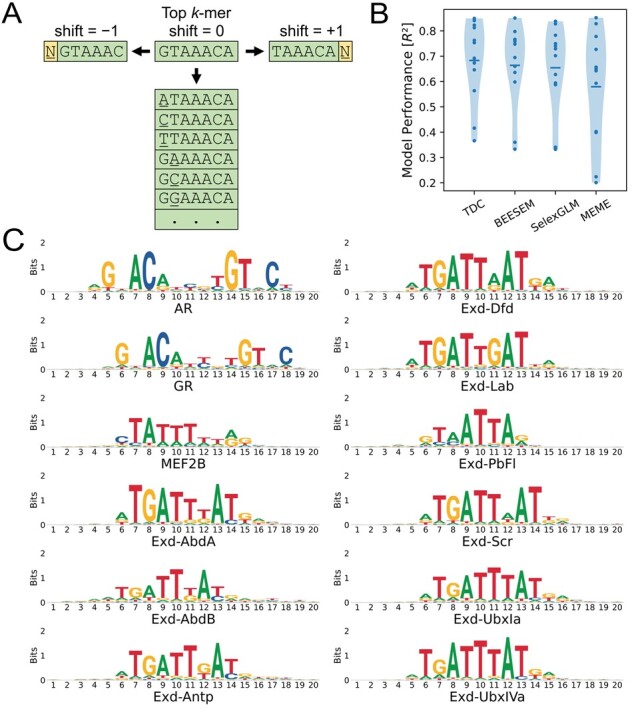
TDC overview and performance. (**A**) Depiction of a single iteration of TDC, showing how the algorithm would align several similar *k*-mers based on a given reference. (**B**) Violin plots showing model performance across 12 SELEX-seq datasets. MLR models were trained using base sequence, minor groove width, and electrostatic potential information along aligned 10-mers to predict the log enrichment of 10-mers with a Z-score larger than 2. Models were trained using 5-fold cross validation with elastic net regularization and the median performance across the tests is reported. (**C**) TDC PWMs generated using all 10-mers aligned with a shift of ±5 bp, weighting each sequence by its relative enrichment

TDC was developed with one goal in mind: the usage of high-affinity sequences to describe the binding of similar, but lower-affinity sequences. Then, use those sequences to align other similar sequences. More specifically, the algorithm starts by assigning the *k-*mer with the largest binding metric a shift of 0 bp and is set as the first reference. All unaligned *k-*mers that are one single bp mutation away from the reference are then added to the alignment and assigned a shift equal to that of the reference (0 in this case). All unaligned *k-*mers overlapping the reference by up to *k*−2 bp are then added to the alignment with a shift equal to that of the reference ±1 or 2 bp depending on if that sequence is overlapping on the 5′ or 3′ end of the reference (Fig. 1A). For example, the sequence AGTAAAC would overlap with the 5′ end of GTAAACA with a shift of −1 bp. After this round, the reference sequence is marked as ‘complete’ and the next reference is determined as the most enriched *k*-mer amongst those which have been aligned, excluding sequences already marked as complete. As before, the new reference is used as a starting point for the addition of more *k*-mers to the alignment, so long as they have not been added previously. This process is terminated when all sequences added to the alignment have been marked as ‘complete’. Detailed pseudocode of this process is made available in Supplementary Table S1.

TDC is provided through a freely accessible webserver and requires only a list of sequences with their corresponding binding measurements as input. After processing the upload, the user is given a summary of the alignment as well as a representative PWM, weighting each sequence by its associated binding metric. This is most appropriate for SELEX-seq data, for which the relative enrichment is expected to approximate the relative binding affinity (Riley *et al.*, 2014; Slattery *et al.*, 2011). The alignment itself is provided as a tab-delimited file where gaps are represented by the ‘_’ character. The output is stored for 48 h and can be accessed using a unique link generated for the submission. TDC can also be run locally as a python package available through pip.

## 3 Results and comparisons

Given a PWM from a motif-generating method such as MEME, BEESEM or SelexGLM, *k-*mers can be assigned to their most likely ‘shift’ relative to the reference, similar to TDC. This is done by padding a given PWM with neutral positions on the 5′ and 3′ ends, then sliding each *k-*mer along every window along the PWM to see which shift results in the highest score. The alignments generated by these methods can then be directly compared with those provided by TDC. A generalizable workflow for PWM-based *k*-mer alignment and evaluation is provided at https://github.com/bhcooper/TDC_evaluation.

MEME is a well-established method for the alignment of sequences, but it does not take quantitative data as input. Therefore, every sequence is weighted equivalently in the construction of the PWM. For this reason, we run MEME using only *k-*mers with a log enrichment two standard deviations above the mean. The resulting PWM can then be used for the alignment of all *k-*mers as described above. Alternatively, BEESEM was made specifically for creating PWMs from SELEX-seq data but is computationally limited to producing motifs no longer than 10 bp and relies on subsampling for particularly large datasets (Ruan *et al.*, 2017). Although SelexGLM is able to generate much longer PWMs (Zhang *et al.*, 2018), the currently available implementation has considerable memory requirements (Supplementary Table S2, Supplementary Fig. S1), and the output depends on the specification of several hyperparameters.

To compare TDC with alternate alignment methods, we include the analysis of 12 SELEX-seq datasets that have previously been published (Abe *et al.*, 2015; Dantas Machado *et al.*, 2020; Zhang *et al.*, 2018). We use a *k-*mer length of 10 bp, which covers the known binding site for most of the TFs considered and contains more information about suboptimal binding sites compared to longer *k-*mers. Although the goal of TDC is alignment rather than PWM generation, we can generate a logo from each alignment, weighting each sequence by its relative enrichment (Fig. 1C, Supplementary Fig. S2). We found the resulting PWMs to be most similar to those generated by BEESEM, with additional information outside the center of the binding site, covering about 15 informative positions for the androgen and glucocorticoid receptor binding sites (Supplementary Fig. S2).

For a more in-depth comparison, we determine what percent of significantly enriched 10-mers are assigned to the same shift, using TDC as the reference. TDC showed a high level of agreement with BEESEM, followed by MEME and SelexGLM (Supplementary Table S3, Supplementary Fig. S1). To evaluate the quality of each alignment, an MLR model was trained to predict the log enrichment of aligned 10-mers which were significantly enriched. Base pairs were one-hot encoded for each position, and the predicted minor groove width and electrostatic potential were included to account for interdependencies between positions (Chiu *et al.*, 2016). For MLR to perform well, sequences need to be aligned such that position-specific permutations along the binding site predictably modulate binding affinity. We found TDC to exhibit the best average performance as measured by the median *R^2^* using 5-fold cross validation (Fig. 1B, Supplementary Table S4). Comparing the wall-clock times, BEESEM was the slowest, requiring hours to complete, whereas TDC only takes seconds (Supplementary Table S5 and Supplementary Fig. S1). SelexGLM was faster than BEESEM but required a large amount of memory in the tests performed (Supplementary Table S2 and Supplementary Fig. S1). The primary reason these methods are more computationally demanding is because they work at the level of the full-length read rather than at the level of the *k*-mer. Since MEME was only used to align *k-*mers with a log enrichment two standard deviations above the means, its wall-clock time was dependent on the number of sequences passing this threshold. While the quickest batch, including 653 sequences, was aligned in about 5 s, the slowest batch, including just 4889 sequences took 18 min, demonstrating a 216-fold increase in wall-clock time for a 7.5-fold increase in the number of sequences aligned (Supplementary Table S5). Finally, we tested the MLR performance of various length *k*-mers aligned with TDC and found the optimal length to be 10 bp (Supplementary Fig. S3).

## 4 Conclusions

Although we demonstrate TDC’s speed and performance using SELEX-seq data, the alignment framework is highly flexible as it only depends on the rank of the sequences provided. This allows for the alignment of binding data from a variety of experimental approaches used today and those that are produced in the future.

## Funding

This work was supported by the National Institutes of Health [grant R35GM130376 to R.R.]; the Human Frontier Science Program [grant RGP0021/2018 to R.R.]; and a USC Provost Fellowship to B.H.C.

*Conflict of Interest*: none declared.

## Supplementary Material

btac653_Supplementary_Data
