# Supplementary material for: Top-Down Crawl: a method for the ultra-rapid and motif-free alignment of sequences with associated binding metrics
Source: Bioinformatics. 2022 Sep 30;38(22):5121–3. doi: 10.1093/bioinformatics/btac653 (PMC9665867; doi:10.1093/bioinformatics/btac653)
Supplement: btac653_Supplementary_Data [file btac653_supplementary_data.pdf]

SUPPLEMENTARY DATA

Top-Down Crawl: A method for the ultra-rapid and motif-free alignment of sequences with associated binding metrics

Brendon H. Cooper[1], Tsu-Pei Chiu[1], and Remo Rohs[1,2,*]

[1]Department of Quantitative and Computational Biology, University of Southern California, Los Angeles, CA 90089, USA

[2]Departments of Chemistry, Physics & Astronomy, and Computer Science, University of Southern California, Los Angeles, CA 90089, USA

*Corresponding author: rohs@usc.edu

Supplementary Table S1: Pseudocode of entire alignment process performed by Top-Down Crawl (TDC).

TDC Algorithm

Input: Table containing standard DNA sequences (A,C,G,T) of equal length in column 1 and binding metrics in column 2

```
df ← dataframe containing binding metrics indexed by sequence

// Average reverse complements or copy value from partner if absent from input
df_rc ← Copy df and reverse complement all sequences
df ← Append df_rc to df, group by index, and save mean for each index

// Initialization
df ← Insert boolean column, isAligned, filled with False, to keep track of sequences which have already
     been added to the alignment
df ← Insert boolean column, wasRef, filled with False, to keep track of sequences which have already
     been used as a reference for adding other sequences to the alignment
df ← Insert integer column, shift, filled with N/A, to keep track of the shift assigned to each sequence
top ← Index of seqeunce with largest binding metric from df
k ← length(top)
df[top][shift] ← 0
df[top][isAligned] ← True
Delete reverseComplement(top) from df

// Continue iterating until all aligned sequences have been used as a reference or until all seqeunces are
   aligned
while (isAligned == True and wasRef == False for any row in df) and (isAligned == False for any row in
     df)
    unchecked ← Subset of df including rows where isAligned == True and wasRef == False
    ref ← Index with largest binding metric from unchecked
    refshift ← df[ref][shift]

    SNPs ← Indices within df that are 1 mismatch away from ref and where isAligned == False
    df[SNPs][shift] ← refshift
    df[SNPs][isAligned] ← True
    Delete reverseComplement(SNPs) from df

```

```
    olap-1 ← Indices within df that overlap with the first k – 1 bases of ref and where isAligned == False
    df[olap-1][shift] ← refshift – 1
    df[olap-1][isAligned] ← True
    Delete reverseComplement(olap-1) from df

    olap-2 ← Indices within df that overlap with the first k – 2 bases of ref and where isAligned == False
    df[olap-2][shift] ← refshift – 2
    df[olap-2][isAligned] ← True
    Delete reverseComplement(olap-2) from df

    olap+1 ← Indices within df that overlap with the last k – 1 bases of ref and where isAligned == False
    df[olap+1][shift] ← refshift + 1
    df[olap+1][isAligned] ← True
    Delete reverseComplement(olap+1) from df

    olap+2 ← Indices within df that overlap with the last k – 2 bases of ref and where isAligned == False
    df[olap+2][shift] ← refshift + 2
    df[olap+2][isAligned] ← True
    Delete reverseComplement(olap+2) from df

    df[ref][wasRef] ← True

df ← Subset of df where isAligned == True
df ← Pad indices of df with "-" based on shift
Output Save df as a table including the padded sequence, averaged binding metric, and shift
```

Supplementary Table S2: Peak memory usage for calculation of enrichment (Riley et al., 2014; Slattery et al., 2011) and TDC, BEESEM (Ruan et al., 2017), SelexGLM (Zhang et al., 2018), or MEME (Bailey & Elkan, 1994) based alignments, evaluated for 12 SELEX-seq datasets (Abe et al., 2015; Dantas Machado et al., 2020; Zhang et al., 2018). We also report the memory requirements for *k*-mer level enrichment calculation, since this a necessary step preceding TDC or MEME based alignment as described in the text. Data is plotted in Supplementary Figure S1.

	Enrichment	TDC + Enrichment	BEESEM	SelexGLM	MEME + Enrichment
**AR**	19 GB	20 GB	63 GB	116 GB	20 GB
**GR**	19 GB	19 GB	55 GB	81 GB	19 GB
**MEF2B**	23 GB	23 GB	31 GB	228 GB	23 GB
**Exd-AbdA**	18 GB	18 GB	15 GB	42 GB	18 GB
**Exd-AbdB**	18 GB	18 GB	20 GB	58 GB	18 GB
**Exd-Antp**	18 GB	18 GB	17 GB	46 GB	18 GB
**Exd-Dfd**	18 GB	18 GB	14 GB	35 GB	18 GB
**Exd-Lab**	18 GB	18 GB	16 GB	47 GB	18 GB
**Exd-PbFl**	12 GB	12 GB	28 GB	110 GB	12 GB
**Exd-Scr**	16 GB	16 GB	15 GB	52 GB	16 GB
**Exd-UbxIa**	17 GB	17 GB	18 GB	68 GB	17 GB
**Exd-UbxIVa**	18 GB	18 GB	16 GB	47 GB	18 GB

Supplementary Table S3: Table of alignment agreements, indicating what fraction of sequences were assigned to the same shift according to TDC and a given method. Data is plotted in Supplementary Figure S1.

	BEESEM	SelexGLM	MEME
**AR**	63%	44%	43%
**GR**	68%	74%	56%
**MEF2B**	86%	85%	57%
**Exd-AbdA**	98%	97%	95%
**Exd-AbdB**	71%	69%	27%
**Exd-Antp**	73%	67%	34%
**Exd-Dfd**	97%	89%	65%
**Exd-Lab**	96%	90%	51%
**Exd-PbFl**	85%	83%	72%
**Exd-Scr**	96%	92%	87%
**Exd-Ubxla**	99%	97%	93%
**Exd-UbxlVa**	100%	98%	99%

Supplementary Table S4: Multiple linear regression (MLR) models were trained using base sequence, minor groove width, and electro-static potential information along aligned 10-mers to predict the log enrichment of 10-mers with a Z-score larger than 2. Models were trained using 5-fold cross validation with elastic net regularization and the median performance across the tests is reported. Data is plotted in Figure 1.

	TDC	BEESEM	SelexGLM	MEME
**AR**	0.84	0.80	0.83	0.83
**GR**	0.76	0.75	0.74	0.78
**MEF2B**	0.56	0.60	0.63	0.40
**Exd-AbdA**	0.67	0.69	0.70	0.66
**Exd-AbdB**	0.37	0.33	0.33	0.22
**Exd-Antp**	0.42	0.36	0.34	0.20
**Exd-Dfd**	0.65	0.64	0.59	0.64
**Exd-Lab**	0.69	0.66	0.59	0.40
**Exd-PbFl**	0.75	0.76	0.75	0.73
**Exd-Scr**	0.82	0.76	0.78	0.65
**Exd-Ubxla**	0.81	0.77	0.73	0.59
**Exd-UbxlVa**	0.85	0.85	0.84	0.85

Supplementary Table S5: Wall-clock time required for each of the methods evaluated. We also report the time requirements for *k*-mer level enrichment calculation, since this a necessary step preceding TDC or MEME based alignment as described in the text. Data is plotted in Supplementary Figure S1.

	Enrichment	TDC + Enrichment	BEESEM	SelexGLM	MEME + Enrichment
**AR**	0h 3m 9s	0h 3m 41s	21h 17m 59s	1h 26m 0s	0h 11m 32s
**GR**	0h 3m 5s	0h 3m 40s	19h 6m 31s	0h 41m 6s	0h 18m 55s
**MEF2B**	0h 4m 1s	0h 4m 28s	10h 6m 30s	4h 46m 34s	0h 11m 56s
**Exd-AbdA**	0h 1m 47s	0h 1m 57s	4h 24m 37s	0h 27m 10s	0h 1m 53s
**Exd-AbdB**	0h 2m 8s	0h 2m 38s	5h 53m 40s	0h 35m 27s	0h 3m 31s
**Exd-Antp**	0h 2m 2s	0h 2m 22s	4h 59m 24s	0h 34m 47s	0h 2m 23s
**Exd-Dfd**	0h 2m 2s	0h 2m 9s	3h 58m 42s	0h 21m 53s	0h 2m 7s
**Exd-Lab**	0h 2m 23s	0h 2m 37s	4h 28m 12s	0h 26m 6s	0h 2m 33s
**Exd-PbFl**	0h 1m 30s	0h 2m 17s	9h 8m 54s	1h 34m 51s	0h 19m 35s
**Exd-Scr**	0h 1m 46s	0h 2m 1s	4h 14m 29s	0h 36m 9s	0h 2m 0s
**Exd-Ubxla**	0h 2m 9s	0h 2m 27s	5h 18m 2s	0h 53m 4s	0h 3m 18s
**Exd-UbxlVa**	0h 1m 51s	0h 2m 4s	4h 31m 49s	0h 27m 14s	0h 2m 2s

Supplementary Figure S1: Violin plots of data given in Supplementary Tables S2, S3, and S5. (Violin plots of data given in Supplementary Table S4 are shown in Figure 1.)

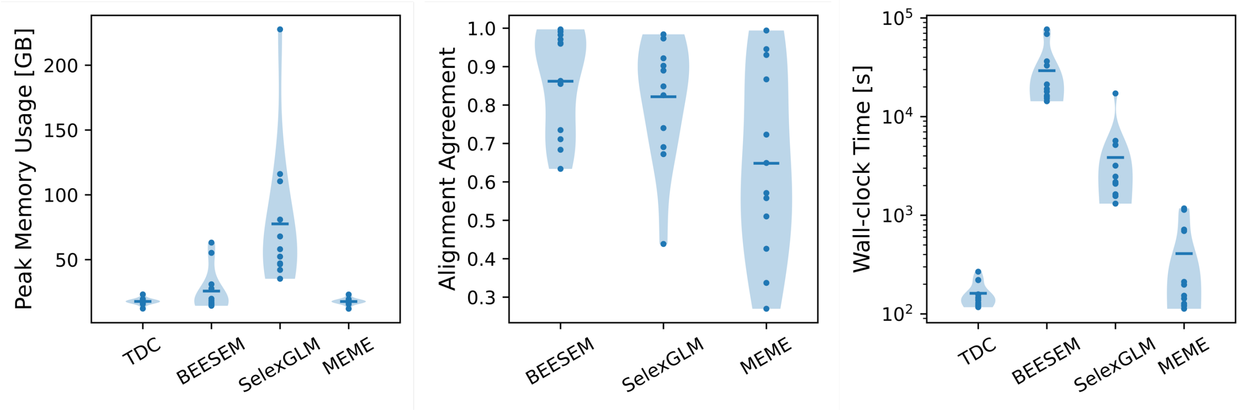

Supplementary Figure S2: Comparison of PWMs generated from each method. The TDC PWM is generated using all 10-mers aligned with a shift of ±5, weighting each sequence by its relative enrichment. The units of the SelexGLM method are provided in terms of $-\Delta\Delta G/RT$ as described in the original method. All others are shown in terms of bits.

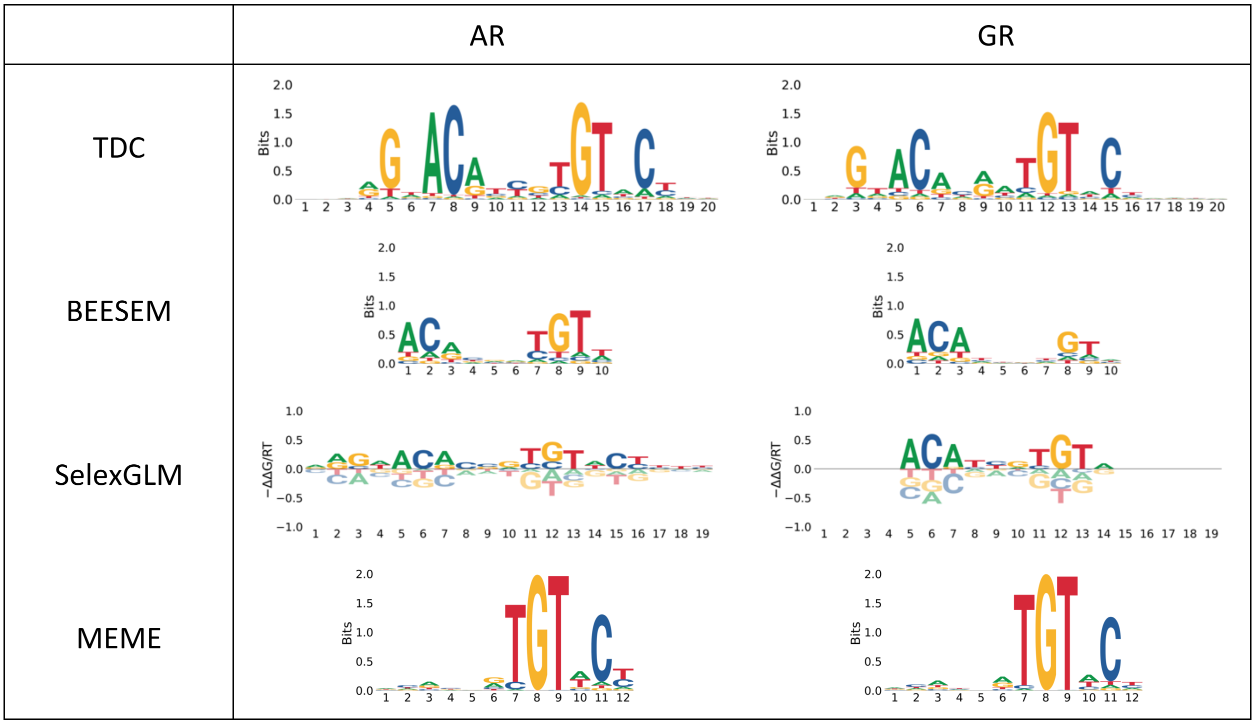

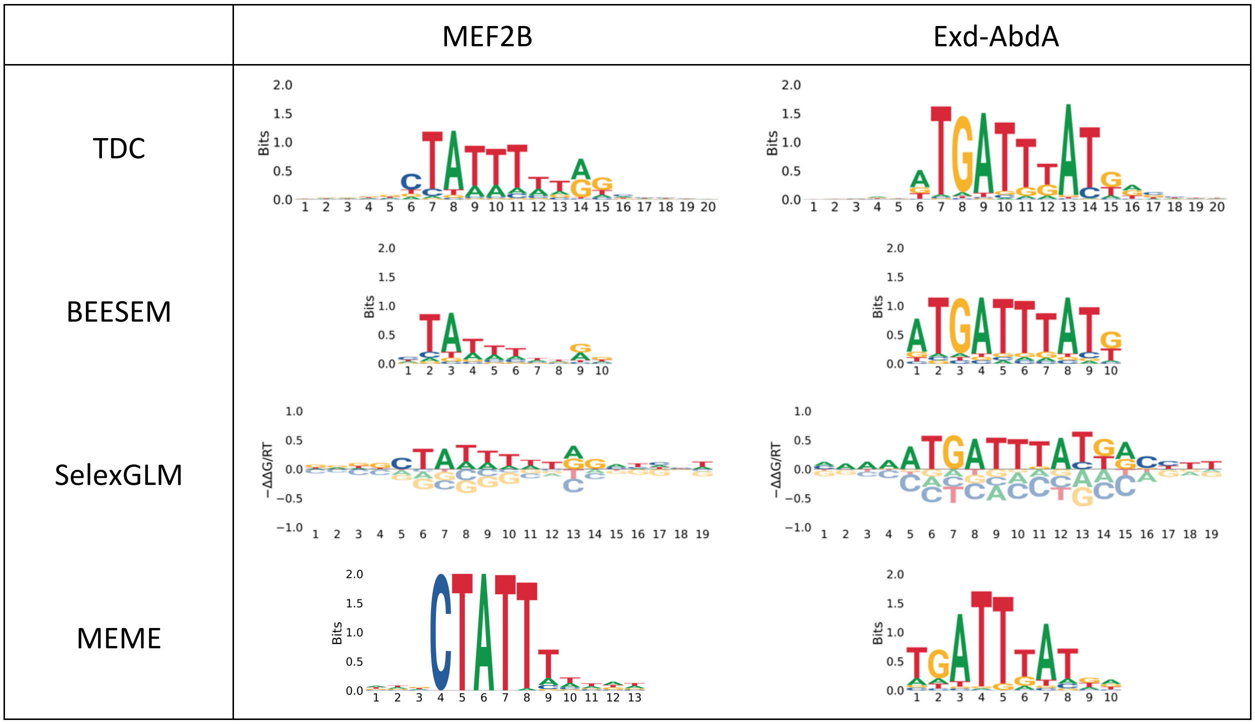

	Exd-AbdB	Exd-Antp
TDC	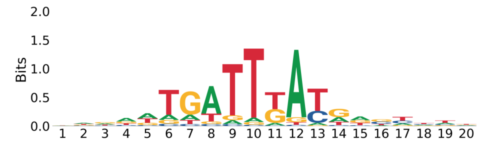	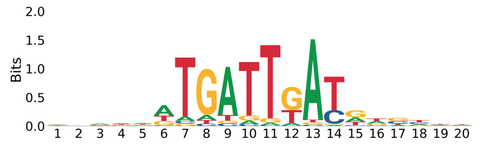
BEESEM	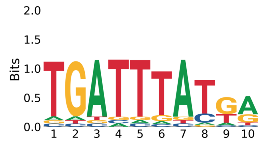	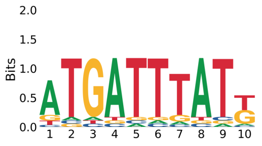
SelexGLM	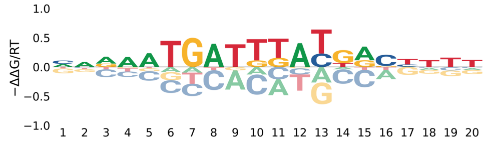	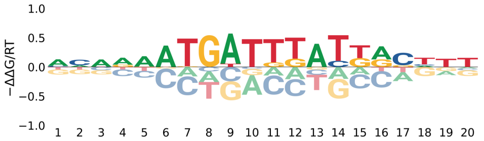
MEME	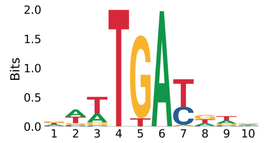	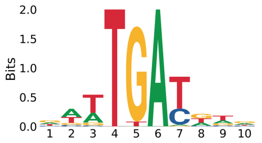

	Exd-Dfd	Exd-Lab
TDC	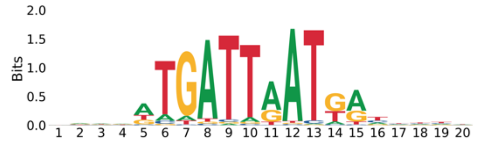	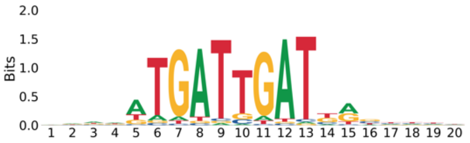
BEESEM	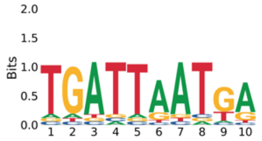	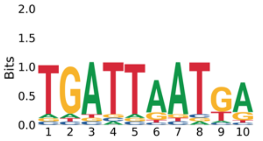
SelexGLM	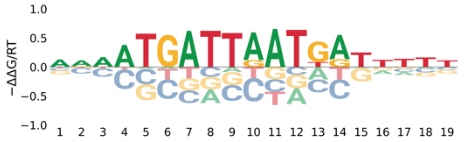	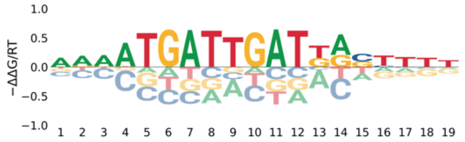
MEME	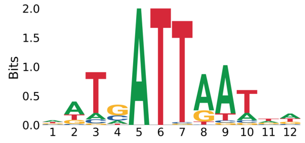	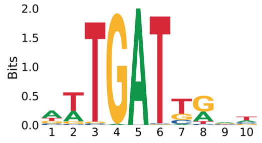

	Exd-PbFl	Exd-Scr
TDC	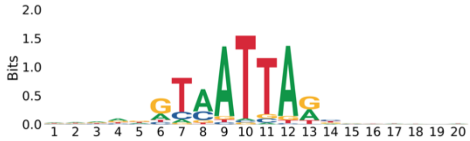	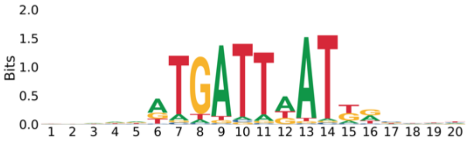
BEESEM	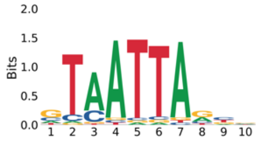	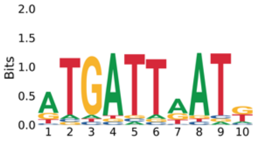
SelexGLM	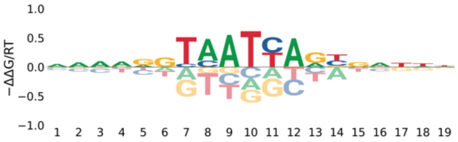	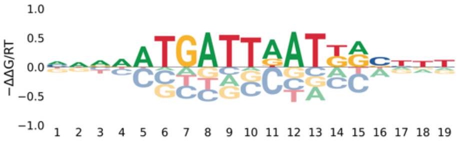
MEME	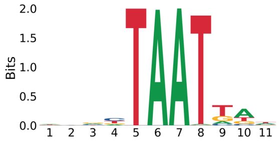	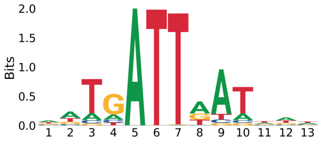

	Exd-UbxIa	Exd-UbxIVa
TDC	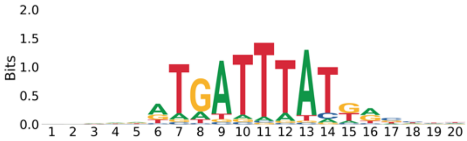	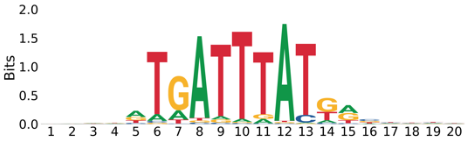
BEESEM	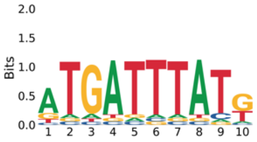	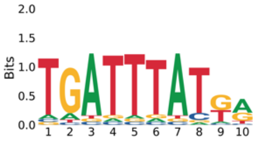
SelexGLM	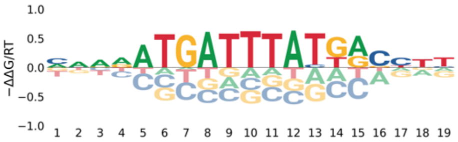	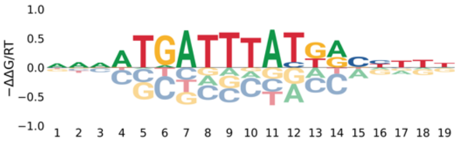
MEME	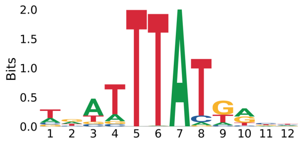	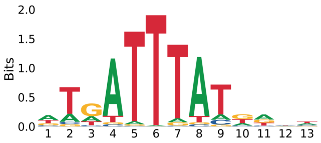

Supplementary Figure S3: Violin plots showing model performance across 12 SELEX-seq datasets, using various length *k*-mers as input to TDC. MLR models were trained using base sequence, minor groove width, and electrostatic potential information along aligned *k*-mers to predict the log enrichment of *k*-mers with a Z-score larger than 2. Models were trained using 5-fold cross validation with elastic net regularization and the median performance across the tests is reported.

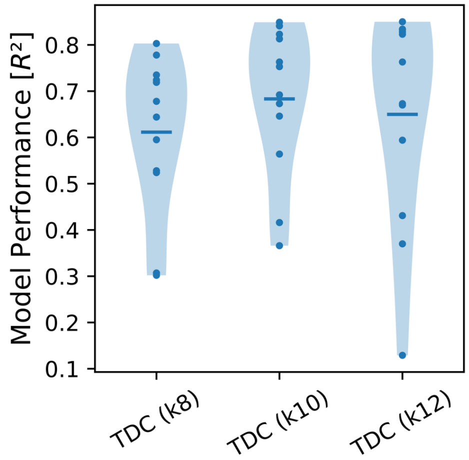

Supplementary References

Abe, N., Dror, I., Yang, L., Slattery, M., Zhou, T., Bussemaker, H. J., Rohs, R., & Mann, R. S. (2015). Deconvolving the recognition of DNA shape from sequence. *Cell*, *161*(2), 307-318.

Bailey, T. L., & Elkan, C. (1994). Fitting a mixture model by expectation maximization to discover motifs in biopolymers. *Proc. Int. Conf. Intell. Syst. Mol. Biol.*, *2*, 28-36.

Dantas Machado, A. C., Cooper, B. H., Lei, X., Di Felice, R., Chen, L., & Rohs, R. (2020). Landscape of DNA binding signatures of myocyte enhancer factor-2B reveals a unique interplay of base and shape readout. *Nucleic Acids Res.*, *48*(15), 8529-8544.

Riley, T. R., Slattery, M., Abe, N., Rastogi, C., Liu, D., Mann, R. S., & Bussemaker, H. J. (2014). SELEX-seq: a method for characterizing the complete repertoire of binding site preferences for transcription factor complexes. In *Hox Genes* (pp. 255-278). Springer.

Ruan, S., Swamidass, S. J., & Stormo, G. D. (2017). BEESEM: estimation of binding energy models using HT-SELEX data. *Bioinformatics*, *33*(15), 2288-2295.

Slattery, M., Riley, T., Liu, P., Abe, N., Gomez-Alcala, P., Dror, I., Zhou, T., Rohs, R., Honig, B., & Bussemaker, H. J. (2011). Cofactor binding evokes latent differences in DNA binding specificity between Hox proteins. *Cell*, *147*(6), 1270-1282.

Zhang, L., Martini, G. D., Rube, H. T., Kribelbauer, J. F., Rastogi, C., FitzPatrick, V. D., Houtman, J. C., Bussemaker, H. J., & Pufall, M. A. (2018). SelexGLM differentiates androgen and glucocorticoid receptor DNA-binding preference over an extended binding site. *Genome Res.*, *28*(1), 111-121.

Author Contributions

B.H.C. conceived the TDC method, independently executed the project, and wrote the manuscript. T.P.C. tested the method and provided advice on implementation. R.R. supervised the project. The authors thank Luigi Manna for help with server setup.
